# The Voice of Parents of Children With a Congenital Anomaly – A EUROlinkCAT Study

**DOI:** 10.3389/fped.2021.654883

**Published:** 2021-11-29

**Authors:** Kristina Garne Holm, Amanda Julie Neville, Anna Pierini, Anna Latos Bielenska, Anna Jamry-Dziurla, Clara Cavero-Carbonell, Ester Garne, Jane Clemensen

**Affiliations:** ^1^H.C. Andersen Children's Hospital, Odense University Hospital, Odense, Denmark; ^2^Department of Clinical Research, Faculty of Health Sciences, University of Southern Denmark, Odense, Denmark; ^3^IMER Registry (Emilia Romagna Registry of Birth Defects), Center for Clinical and Epidemiological Research, University of Ferrara and Azienda Ospedaliero-Universitaria di Ferrara, Ferrara, Italy; ^4^Tuscany Registry of Congenital Defects (RTDC), Institute of Clinical Physiology, National Research Council, Pisa, Italy; ^5^Polish Registry of Congenital Malformations, Chair and Department of Medical Genetics, University of Medical Science, Poznan, Poland; ^6^Rare Diseases Research Unit, Foundation for the Promotion of Health and Biomedical Research in the Valencian Region, Valencia, Spain; ^7^Paediatric Department, Hospital Lillebaelt-Kolding, Kolding, Denmark; ^8^Centre for Innovative Medical Technology, Odense University Hospital, Odense, Denmark

**Keywords:** caregiver, congenital anomalies, child, family, communication

## Abstract

EUROlinkCAT aims to investigate the health and educational outcomes of children with congenital anomalies for the first 10 years of their lives. We also aim to facilitate the development of a more reciprocal relationship between families with children with congenital anomalies, health and social care professionals, and researchers by conducting focus groups. The aim of the focus groups and parent interviews was to investigate parental experiences of having a child with a heart defect requiring surgery, cleft lip, spina bifida or Down Syndrome and to identify their research priorities. In total, seven interviews with 12 parents and eight focus groups with 58 parents and two caregivers were conducted in four European countries. We found that parents request more positive information with a focus on quality of life and what the children can achieve rather than solely on the negative aspects and limitations of the congenital anomaly. Some parents also highlighted discrepancies between the family's need for support and the lack of support received from the local authority. Finally, it was challenging for the parents to address specific research priorities. Future research should therefore focus on the potential of a child with a congenital anomaly.

## Introduction

Congenital anomalies are a major contributor to infant and childhood morbidity and mortality (1–3). Major congenital anomalies affect one in 50 live births (4) with congenital heart defects being the most frequent (4). Survival of children with congenital anomalies has improved (5), so up-to-date knowledge about childhood morbidity is required in order to counsel parents both after a prenatal or postnatal diagnosis. With increasing survival, more families are caring for infants and children with congenital anomalies and face many everyday challenges (6–8). EUROlinkCAT is a 5 year research project (9) with the aim to establish a linked European cohort of children with congenital anomalies partnering with EUROCAT (European Surveillance of Congenital Anomalies in Europe) registries (10). EUROlinkCAT aims to expand the knowledge on the survival, health and education of children in Europe born with major congenital anomalies and to investigate health inequalities. Previous studies of parental involvement in health research have identified issues that clinical researchers may not be aware of (11) and EUROlinkCAT acknowledges that parents have extensive knowledge on their child's symptoms, the health care system and challenges occurring in everyday life.

To incorporate parental needs and expectations into the healthcare system and define research priorities their involvement is imperative. Little is known about parental involvement in health research, despite the increasing interest of doing so (11). The part of the EUROlinkCAT project reported here aims to investigate parent's experiences, needs and wishes both for improvement of the collaboration between parents and the healthcare system and for future research. Thus, we want to take parent involvement to a new level by inviting them to participate in a large research project across EU countries.

The EUROlinkCAT project aims to close the gap between research and clinical treatment, and to ensure evidence-based practice where parent's knowledge and perspectives are included. This study provides new knowledge to be used to leverage changes in clinical practice incorporating parents' needs and help researchers follow parental priorities for conducting future research studies.

The aim of the study was to investigate parental experiences of having a child with one of four congenital anomalies: Down syndrome, spina bifida, cleft lip or severe congenital heart defects requiring surgery. We asked parents questions in two key areas: “What is important for you having a child born with a congenital anomaly? And what should the researcher focus on in upcoming projects?”

## Methods

### Study Design

Due to the aim of investigating parental experiences of having a child with a congenital anomaly we conducted a qualitative approach. The qualitative approach gave us the opportunity to hold an holistic position and obtain in-depth knowledge about the parent's experiences and their research priorities (12). To obtain the needed information from the parents we conducted interviews and focus groups. The interviews were conducted first in order to identify what parents experience when living with a child with one of the four congenital anomalies and to understand their research priorities. Secondly, the interviews were carried out to test and qualify the interview guide for focus groups. The interviews identified challenges when talking with the parents about their research priorities.

### Sample

As the total group of congenital anomalies included in the EUROlinkCAT study is very heterogeneous we decided that parents of children with four pre-defined congenital anomaly groups with different health problems covering intellectual disability, physical disability, visible defects and non-visible defects with higher mortality, would be included. The four anomaly groups selected were congenital heart defects requiring surgery (non-visible defect with higher mortality), cleft lip (visible defect), spina bifida (physical disability) and Down syndrome (intellectual disability and often associated with congenital heart defect). Besides the four criteria for health problems the anomaly groups were chosen with consideration of not being too rare, resulting in having challenges with inclusion.

### Recruitment

Parents were recruited *via* five EUROCAT registries in Denmark, Spain, Poland, and Italy: Funen County Registry in Denmark, Valencian Region Registry (Registro poblacional de Anomalías Congénitas de la Comunitat Valenciana) in Spain, Wielkopolska Registries in Poland, and Emilia- Romagna Registry (Indagine Malformazioni congenite in Emilia Romagna-IMER) in Italy, Tuscany Registry (Registro Toscano Difetti Congeniti-RTDC) in Italy.

Participants were consecutive recruited through an invitation either from the collaborating hospital physicians or through patient associations for the included anomalies. Inclusion criteria was being biological parent or grandparent of a child aged between one and 10 years old with one of the selected congenital anomalies. Participants had to be able to speak the local language of their country or English. All parents participating in the interviews and focus groups had been regularly in contact with a hospital due to their child's diagnosis. The rationale for choosing Denmark, Spain, Poland and Italy was to have as broad a perspective as possible in relation to culture, organization of health system and social circumstances as possible.

### Data Collection

Semi-structured interviews were conducted in the Region of Southern Denmark by PhD KGH and Professor JC. The Spanish focus group with parents of children with Cleft Palate and Cleft lip was conducted in a meeting room in Barcelona. The focus group was conducted in Barcelona because of a close collaboration between clinicians in Barcelona and the researchers in the Valencian registry. One focus group in Poland with parents of children with Down syndrome was conducted in at the Department of Medical Genetics, Poznan University of Medical Sciences. The other Polish focus groups with parents of children with Spina Bifida and Cleft lip took place in SWPS University of Social Sciences in Katowice and The Specialist Children's Hospital in Olsztyn, respectively. In total, four focus groups were conducted in Italy; for parents of children with Down syndrome a focus group took place in the Trisomy 21 Onlus Association in Florence. The focus group for parents of children with cleft lip was held at St Anna Hospital in Cona Ferrara, and for parents of children with spina bifida the focus group was held at Hotel Parma & Congressi in Parma. Finally, a focus group with parents of children with congenital heart defect took place in the Heart Hospital “Gaetano Pasquinucci” in Massa.

Prior to each interview and focus group, the parents were introduced to the participating researchers and the aim of the study. The focus groups were carried out by researchers from the local EUROCAT registry; BCs AN and BCs AP in Italy and MSc AJ and Professor ALB in Poland. In Spain the focus group was carried out by researchers from the local EUROCAT registry and sociologist Lucía Páramo. EUROlinkCAT produced an online focus group manual to allow researchers to prepare and train for the focus groups. This would also support rigor during the study. There was no relationship between the researchers and the participants prior to the interviews and focus groups. One researcher interviewed and the second researcher took field notes during the focus group. Initially, an interview guide was developed for the interviews in Denmark based on the literature and according to the study aim. As it was challenging for the parents to answer questions about research priorities we added follow-up questions to the interview guide for the interviewers to use during the focus groups to support talking and discussion. The themes in the interview guide included: parents' experience of the pregnancy, birth, daily life and, priorities for future research. To support the questions raised by the interviewer during the focus groups, cards with the themes were visual for the parents to ensure focus on each theme.

The interviews in Denmark were conducted between March and July 2016 and the focus groups in Spain, Poland and Italy were conducted between February 2018 and November 2019. All interviews and focus groups were audio-recorded and transcribed by the researchers from each registry and translated into English. The objective field notes from the focus groups were used during transcripts of the interviews, to help subsidize parental statements (i.e., father crying). The parents participating in interviews in Denmark were provided with the interview transcript after the interviews and were encouraged to read it and provide feedback if the written material did not coincide with their views. None of the parents provided feedback to the transcripts. Of ethical reason, parents participating in focus groups were not provided with interview transcripts while they contained data from other people than themselves.

### Analysis

Data were analyzed using systematic text condensation (STC), which is inspired by Giorgi and adjusted by Malterud (13). STC is a method suitable for descriptive analysis of phenomenon for the development of new descriptions and concepts. A STC analysis consists of decontextualisation and recontextualisation of the data. STC is a procedure of four steps: (I) read transcripts repeatedly to identify themes, (II) identify and code units of meaning, (III) identify sub-groups of codes from step II and develop condensates from them and, (IV) describe experiences based on the condensates (14). To have multiple perspectives during the first process of the analysis, KGH and JC individually extracted themes from data in interview and focus group transcripts. Hereafter themes were triangulated in a face-to-face process where each researcher presented identified themes and from where they derived in data (interview transcripts). JC and KGH reached consensus of themes and presented the themes for the entire research group which agreed. KGH and JC completed steps II-IV, presented the full analysis for the research group to have their comments and finally, all researchers approved the analysis and the presentation of findings.

### Ethics

In Denmark the study was approved by the Danish Data Protection Agency (2008-58-0035) and by the hospital management.

In Italy, after presenting the study to the Regional Health Authority, hospital board, and university it was confirmed that this work was considered a normal part of the registries activities and that specific approval was not required. However, the IMER Registry and the RTDC Registry operate under law decree, which does not allow parents of children in the registry to be contacted directly, so therefore recruitment was conducted through clinicians and parent/patient organizations of children with the condition.

In Poland there was no obligation to have specific ethical approval for this work, as organizing focus groups is one of the tasks of EUROlinkCAT overall which had already received ethics approval.

In Spain there was no obligation to have specific ethical approval for this work, as organizing focus groups is one of the tasks of EUROlinkCAT overall which had already received ethics approval.

According to the Helsinki declaration (15) the parents in the four regions received written and oral information about the study. The parents gave written informed consent to participate prior to the interviews and focus groups. The parents could withdraw from the study at any time with no consequences for the future treatment of their child or themselves.

## Results

A total of 12 parents participated in seven interviews and 58 parents and two caregivers participated in eight focus groups (Table 1). The interviews lasted between 55 and 104 min. The focus groups lasted between 180 and 200 min. Overall, the analysis identified no notable differences in parent's experiences and expectations identified in the interviews and focus groups across the four countries.

**Table 1:** Participants in interviews and focus groups.

	**Mother, *n***	**Father, *n***	**Caregiver, *n***
**Individual interviews**			
Down syndrome, Denmark	1		
Spina bifida, Denmark	2	2	
Cleft lip and palate, Denmark	2	2	
Congenital heart defects, Denmark	2	1	
**Total**	**7**	**5**	
**Focus groups**			
Cleft lip and palate, Poland	7	1	
Spina Bifida, Poland	7	4	1
Down syndrome, Poland	4		
Down syndrome, Italy	5	3	
Spina bifida, Italy	3	2	
Cleft lip and palate, Italy	7	3	1
Congenital heart defects, Italy	4	2	
Cleft palate, Spain	6		
**Total**	**43**	**15**	**2**

Analysis of the interviews and focus groups identified two overall themes (Table 2); *The challenges of tailored information* and *Parenting in between compassion and bureaucracy*.

**Table 2:** Systematic text condensation of transcripts.

**Themes**	**From themes to codes**		**Sub-categories**	**Overall categories**
	Quotes	Codes		
				**The challenges of tailored information**
Presentation of diagnosis	*She (the Doctor) said all the worst things about spina bifida* (mother, spina bifida, Poland)	The parents expressed that they felt clinicians had a tendency to only present the worst case scenario of the anomaly.	Discrepancy	
Support	*I could have used spending time with other mothers with disabled children* (mother, down syndrome, Italy)	Parents feel that it is important to have contact with other parents experienced in parenting a child with an identical anomaly to hear the good stories.	Searching for peers	
Empathy	*It is very important with human relationship* (father, cleft lip, Italy)	Some parents perceived a lack of empathy from the clinicians.	Compassion	
				**Parenting in between compassion and bureaucracy**
The child	*We live in the countryside and love the forest and we wanted him to be able to join our walks in the forest. When he was two, he managed the ATV all by himself. He should not be limited* (father, spina bifida, Denmark)	The parents expressed that they wanted to support their child in their development.	Parental love	
Parental experiences	*So much money had been spent on saving his life, but after his discharge the municipality would not help us* (mother, congenital heart disease, Denmark)	One of the major challenges the parents experienced in daily life with their child was the bureaucracy when applying for support or help supplies.	Fighting battles	
Surroundings	*So it's something that I believe you see in people's faces. And I imagine the lip, which is more of a cosmetic thing, people are not very tactful and I've heard them say terrible things* (mother, cleft palate, Spain)	Parent and children with visible anomalies experienced being starred at. Parents perceive that people often don't know what to say or do leading to hurtful reactions or comments.	Being exposed	

### The Challenges of Tailored Information

A recurrent observation from the analysis was that parents that received a prenatal diagnosis of the congenital anomaly were happy they had received it before the child was born so they could prepare themselves. However, the parents that did not know about their child's diagnosis before birth were happy that they did not know prior to the birth of their child. A father stated that he could not bear the thought of having chosen an abortion if they had known about the anomaly before birth.

Around the time of diagnosis, regardless if it was prenatal or at birth, the parents were very reliant on open and trustworthy communication with clinicians. When clinicians engage in an open dialog and signal sincere empathy, with both spoken- and body language, the parents perceived the information about the diagnosis was given with a sense of trust. Further, parents feel acknowledged when communication occurs parallel to receiving compassion and care from the nurses. However, some parents in this study did not experience such compassion from their clinicians.

*“He [the clinician] explained the analysis and said that I was affected by a fetus with…. as if it was cancer to be removed”* (mother, Down syndrome, Poland).

Some of the parents felt that information provided by the clinicians was not neutral but colored by the clinicians own beliefs. Parents found this unprofessional. Most parents felt dependent on clinicians, creating difficulties where clinicians have opinions that the parents did not share. This was identified by the discrepancy between the information the clinicians provided and the information the parents requested. Many of the parents of children with Down syndrome, spina bifida and cleft lip perceived that the clinicians in the hospital focus too much on the physical malformation/anomaly instead of all the other aspects that define a human being. Clinicians provided information concerning what the child would not be capable of due to his/her congenital anomaly instead of focusing on the competences and the potential for development that the child could have. A few parents stated they wanted more positive information about what their child would be capable of instead of only being told what their child would never be capable of.

*“….the doctor immediately describes what the worst scenario is. He doesn't tell their potential”* (mother, spina bifida, Italy)

The parents with children with severe congenital heart defect requiring surgery experienced the information around the diagnosis as balanced. When the parents felt sufficiently informed about the diagnosis they did not search for further information. However, parents who did not feel sufficiently informed searched for more information turning to social media and Google. Thus, today's parents do search the internet for information, but there are two dimensions of internet searching after having a child with a diagnosis. It can be a source of lots of information, but also *an ugly beast* as one mother described it. When doing internet searches, there was no filter helping the parents interpret the information. To obtain more information and have a broader perspective on their child's diagnosis, the parents reached out to other parents, either through Facebook groups or patient associations.

*“The best contact is with a parent, not a psychologist”* (mother, Down syndrome, Italy).

Some parents withdrew their membership from diagnosis specific Facebook groups because there was too much focus on the negative aspects of the diagnosis. The parents differed according to when and why they needed to get in contact with peers, but all needed the contact at some point. Two parents described that they did not have the strength to engage with other parents until a year after the diagnosis, even though they felt insufficiently informed and lacked trust in health care professionals. Their home health nurse then helped them engage with peers. On the other hand, some parents asked for peers right after birth. One mother described that she gave birth to her child and later discovered that another woman had given birth to a child with Down syndrome at the same ward at the same time, but no one thought about connecting them: *“It would have been a great opportunity to talk and support each other*” (mother, Down syndrome, Italy). Some parents had not yet met with other parents.

The parents participating in the congenital heart defect focus group expressed the need for support from a psychologist. It was challenging for these parents not knowing the outcomes of their child's surgery as the doctors were unable to fully guarantee a successful outcome. These parents felt unsure whether they would see their child alive again, which was emotionally exhausting. The parents also requested psychological support for siblings.

### Parenting in Between Compassion and Bureaucracy

Everyday life for the parents is filled with infinite joy for their children. The parents participating in interviews and focus groups concentrated on quality of life for their children and possessed the belief that physical or cognitive impairment is not equivalent with low quality of life. After the new-born period, and when family life with new routines has settled, the parents work hard to support the development of their child. As the child gets older, challenges for the child with physical and/or cognitive impairment increase and it becomes more visible to the parents that their child is different from children without a congenital anomaly. Despite the child's challenges, the parents said they think about opportunities rather than limitations. Parents with children with Down syndrome or spina bifida know that training is essential for development of competences for their child. However, the many of the parents perceived lack of support from their clinician.

*“I was told from a doctor that these children are very difficult and I shouldn't expect anything from him. That was awful, how could he say that?”* (mother, Down syndrome, Italy)

Parents of children with cleft palate described challenges with feeding their child and lacked information and practical guidance. As one mother describes the information, she gained from a doctor:

*“Feed the baby in an upright position and in case she chokes, perform basic resuscitation*. *And after a year I'll operate on her and she'll be as good as new”* (mother, cleft palate, Spain)

In this case, the mother felt insecure and was overwhelmed with the fact that surgery was not within a year. Many parents perceived to be left alone with the feeding challenges resulting in emotions of guilt and anger.

Experiences of guilt and blame appeared in more ways. The mothers blamed themselves for their child's anomaly and kept reflecting on their behavior, medication and nutrition in the weeks where the anomaly typically arose. Further, parents described situations where they were questioned why they continued the pregnancy. Finally, some parents of children with cleft lip had experienced people asking them to stay indoor with their child not to frighten the public with the appearance of the child.

Fighting for support was a common theme among the parents. Some parents expressed that the worse part of having a child with a congenital anomaly, and thereby special needs, is the constant battle with the local authorities. Parents of children with congenital heart defects who often suffer infections found it difficult to manage their jobs. Some parents had to work less hours and others had to quit their jobs entirely resulting in financial pressure for the family.

*“So much money had been spent on saving his life, but after his discharge the local authority would not help us. I had to quit my job, because he was always sick…. and ended up as divorced.”* (mother, congenital heart defect, Denmark).

In general, the majority of parents lack support from the local authority to cope with their family‘s situation even though they may have a legal right (for example for special schooling or reduced working hours). Parents find fighting battles with the local authority for support is very resource demanding and emotionally draining. When parents' expectations for sufficient help for training their child exceeded the offers provided from hospital or local authority, they wanted to train their child themselves. However, it was not always possible for the parents due to abilities, resources and economic obligations.

*“I would love to work part-time so I could increase intellectual training with her, because studies show that these children can learn. But I'm not supported in that perception.”* (mother, Down syndrome, Denmark).

Battles with the local authorities are also demanding and requires a lot of competences, stubbornness, and time. Time that the children could have benefitted from. All parents expressed that they repeatedly applied for extra hours of work or other forms of help, but their requests often resulted in rejection after rejection. Parents felt at times that they had to act as a lawyer protecting their child's rights.

*“To be Honest, We Take Care Privately Because My Patience With the Whole System Is Running out”* (Father, Spina Bifida, Italy)

The parents felt that these interactions with the local authority were the worse part about having child with a congenital anomaly. The lack of support that parents received led to parents believing that their child is not acknowledged as a human being with potential.

When questions concerning the parent's wishes and priorities for future research were raised, it was challenging for the parents to give specific answers. However, we identified that the research priorities for the parents were around quality of life (for all four anomaly groups), cognitive development (Down syndrome, spina bifida, and congenital heart defects), and how much training results in improved cognitive and physical outcome (for all four anomaly groups).

## Discussion

The overall aim of this qualitative study was to investigate parental experiences of having a child with one of four congenital anomalies: Down syndrome, spina bifida, cleft lip or severe congenital heart defects requiring surgery. Despite cultural differences and different hospital settings, the parents had the same worries, identical expectations and experiences with information from clinicians.

### Holistic and Empathic Communication

The research questions identified that informing parents of their child's condition, both pre- and postnatal, requires careful presentation of the diagnosis and its impact on the child's life. Studies concerning parental perspectives about being informed of an anomaly in their child have been widely studied (16–20) and recommendations for information strategies have been presented when a congenital anomaly is diagnosed prenatally (21) and postnatal (22). We identified that parents requested more holistic information about the diagnosis and the prognosis taking the child's abilities and future possibilities in consideration. This is identical with the findings from Davies et al. (23), who identified that when health care providers focus on the positive when interacting with parents of children with complex and chronic conditions it resulted in positive outcomes for both parents and health care providers. However, physicians are legally obliged to inform about the risks of the condition and potential short and long-term complications of planned procedures and surgeries (24) and this does not always correlate with what the parents want to hear.

We also identified that some parents in this study perceived that they lacked empathy from physicians providing information about their child's anomaly and they therefore searched for more information from associations, peers, and the Internet. Empathy has been identified as one of the main elements when interacting with parents with ill children (23). Hilton-Kamm identified that parent's perception of the physician's compassion and empathy when providing information about a congenital heart defect diagnosis is inversely related of the parents seeking second opinions (25) and that they request more information during the perinatal and neonatal period than physicians provide (26).

### Parental Concerns

We also identified that having a child with a congenital anomaly leads to numerous concerns. It was clear from the interviews and focus group discussions that the parents fully accept the disabilities of their child and fight for the rights of their child. Another study of parents expecting a child with a congenital anomaly has identified that the parents already during pregnancy focus on characteristics and strengths rather than disabilities or obstacles (27). The parents in our study were generally more concerned about long-term prognosis and quality of life than of the risk of surgery in infancy. This correlates with the objective of EUROlinkCAT with examine morbidity and education to 10 years of age. The findings from this study indicates that parents experiences challenges and they appear both in hospital- and community settings. Many clinicians and sectors are involved when a child is born with a congenital anomaly and this continues throughout childhood. To increase parent support cross-sectoral collaboration must be prioritized to ensure their needs.

### Research Priorities

What the parents requested was research on how parents can increase the quality of life for their children and what the parents can do to optimize the long-term outcome for their child. A case study has shown that specific training of children with Down syndrome increased motor and cognitive development (28), but to our knowledge the impact on the amount of parental training has not been studied in relation to long-term motor and cognitive outcome as well as the impact on quality of life. Carlsson et al. have involved couples who experienced a prenatal diagnosis of a congenital heart defect to assess their perspectives on research priorities (29). They found that their participants requested more research on written material, similar to the parents in our study. For counseling parents after a diagnosis of a congenital anomaly, holistic up-to-date information on short and long-term outcomes are needed. Finally, the parent's research priorities focused on long-term outcomes and quality of life as important themes.

### Implications for Clinical Practice

Our findings indicate that encouragement and compassion can be the keys to give the family a better start with their new baby. Thus, we suggest that clinicians working with these families meet them with kindness and compassion focusing on the child's possibilities and abilities. Further, the clinicians must see the family's needs in a holistic perspective involving support persons from primary care and local authorities in general. Finally, we suggest more research focusing on the family's needs and wishes.

### Methodological Considerations

A limitation to consider is that data were collected in five regions in four European countries. It was planned that focus groups should be carried out with parents of all four anomaly groups in the participating countries and further, more focus groups were planned in other European countries (Malta, Portugal and England). However, the COVID-19 situation in Europe haltered the continuation for the focus groups. This study expresses the opinions of parents with live children whose views may be very different from parents who decide termination of pregnancy due to fetal anomaly. Our initial expectation was that there would be differences across the countries in parental experience and perceptions due to cultural and social circumstances, and organization of health systems. Further, another limit concerns the multi-dimensional heterogeneities that exist in congenital anomalies. This might be particularly present in the CHD group with different heart defects requiring different clinical management and prognosis of the children. It was a requirement for inclusion that surgery was performed to avoid inclusion of less severe heart defects. However, we mainly identified similarities.

Parents of children up to 10 years of age were included in the study why recall must be considered. Further, clinical practice and the approach to parents of children with congenital anomalies have developed during the last decade.

During the interviews and focus groups we identified that it may be challenging for people who are not familiar with research to identify research possibilities and limitations. It requires help during the interviews to encourage the parents to express their priorities for future research.

Parents in the study lead challenging everyday lives so we expected last minute dropouts and included a minimum of ten parents for each focus group. For two focus groups we experienced last minute drop-outs from parents and therefore two focus groups were carried out with less than the recommended number of participants (13). However, we did not experience that it negatively affected the dynamic and discussion among the participants. No parents participating in interview or focus groups withdraw from the study. All parents participating expressed their satisfaction with participation in a focus group and the opportunity to release their emotions, say their opinion, and share their experiences with other parents. Parents communicate with each other *via* social media, but face to face contacts in small groups are rare. The energy and insights generated by the focus groups will be used for further involvement of parents and health professionals. All parents were offered to contact the researchers after interviews and focus groups if in need for a more personal talk. The focus groups were facilitated by the strong working relationships between EUROCAT Registry leaders, clinicians and patient associations with which they have long-standing relationships. The research group is interdisciplinary consisting of physicians, nurses, and epidemiologists from different countries. This provided the group with different perspectives and pre-understanding. All researchers took active part in collecting data. A common platform for training of the researches for conducting focus groups, an interview guide and a template of how to transcribe data where developed to minimize the effect of individual beliefs and assumptions.

## Conclusion

The parents request more positive information instead of solely focusing on the negative aspects of living with the anomaly regardless if the child is diagnosed during pregnancy or at birth. It was challenging for the parents to directly address specific research priorities, but we identified that the parents had great concerns about their children's quality of life and cognitive and physical achievements and were caught between seeking the best possibilities for their child and the limited resources that the public system can provide.

## Data Availability Statement

Data supporting the conclusions of this article can be made available by the authors upon request.

## Ethics Statement

The studies involving human participants were reviewed and approved by EUROlinkCAT under the Danish Data Protection Agency (2008-58-0035). Written informed consent was not required for the study on human participants, in accordance with the local legislation and institutional requirements.

## Author Contributions

KH: methodology, investigation, formal analysis, writing original draft, and writing review and editing. AN: investigation, resources, visualization, supervision, and writing original draft. AP and AL: investigation and resources. AJ-D: investigation. EG: supervision, project administration, and funding acquisition. CC-C: investigation and writing review and editing. JC: conceptualization, methodology, formal analysis, investigation, writing original draft, and writing review and editing. All authors read and approved the final version of the manuscript.

## Funding

This project had received funding from the European Union's Horizon 2020 research and innovation programme under grant agreement No 733001.

## Conflict of Interest

The authors declare that the research was conducted in the absence of any commercial or financial relationships that could be construed as a potential conflict of interest.

## Publisher's Note

All claims expressed in this article are solely those of the authors and do not necessarily represent those of their affiliated organizations, or those of the publisher, the editors and the reviewers. Any product that may be evaluated in this article, or claim that may be made by its manufacturer, is not guaranteed or endorsed by the publisher.
